# Aurora kinase B as a therapeutic target in HPV-induced cervical cancer: mechanisms and future perspectives

**DOI:** 10.3389/fonc.2026.1857128

**Published:** 2026-07-03

**Authors:** Medha Karnik, Preethi G. Anantharaju, Arati Sharma, Olga Sukocheva, Edmund Tse, SubbaRao V. Madhunapantula

**Affiliations:** 1 Center of Excellence in Molecular Biology and Regenerative Medicine (CEMR) Laboratory (A DST-FIST Supported Center and ICMR-Collaborating Center of Excellence), Department of Biochemistry (A DST-FIST Supported Department), JSS Medical College, JSS Academy of Higher Education & Research (JSS AHER), Mysore, Karnataka, India; 2 Department of Molecular and Precision Medicine, Center for Cannabis and Natural Product Pharmaceuticals (CCNPP), Penn State Cancer Institute, Hershey, PA, United States; 3 Department of Gastroenterology and Hepatology, Royal Adelaide Hospital, Central Adelaide Local Health Network (CALHN), Adelaide, SA, Australia; 4 Faculty of Health and Medical Sciences, The University of Adelaide, Adelaide, SA, Australia; 5 Special Interest Group in Cancer Biology and Cancer Stem Cells (SIG-CBCSC), JSS Medical College, JSS Academy of Higher Education & Research (JSS AHER), Mysore, Karnataka, India

**Keywords:** alisertib, Aurora kinase B, barasertib, cervical cancer, E6 and E7, HPV

## Abstract

Aurora kinase B (AURKB) is a core component of the chromosomal passenger complex, which plays a central role in regulating chromosome condensation, spindle checkpoint function, and cytokinesis. Dysregulated AURKB activity leads to chromosomal instability and aneuploidy, which are the key drivers of oncogenesis. In cervical cancer, persistent infection with high-risk human papillomaviruses (HPV16 and HPV18) initiates carcinogenesis through the viral oncoproteins E6 and E7, which disable tumor suppressor pathways. Recent evidence indicates that E6 and E7 also influence AURKB activity, thereby exacerbating genomic instability, overriding cell-cycle checkpoints, and accelerating tumor progression. Overexpression of AURKB has been reported in cervical cancer and correlated with tumor stage, therapeutic resistance, and poor prognosis. Preclinical investigations demonstrate that pharmacological inhibition of AURKB suppresses tumor cells proliferation, induces mitotic catastrophe, and enhances sensitivity to chemotherapy and radiotherapy. Although clinical evaluation of these AURKB inhibitors such as barasertib (AZD1152) and AZD2811 remains limited, early findings support their potential efficacy, particularly in rationally designed combination regimens. This review describes the mechanistic interplay between HPV oncogenes and AURKB, highlights its role as a biomarker of aggressive disease, and critically assesses the therapeutic promise of AURKB inhibition. Finally, we outline future perspectives on integrating AURKB-targeted therapies into precision oncology for HPV-driven cervical cancer.

## 1 Introduction

Cervical cancer (CC) continues to pose a major health burden globally. Ranked as the fourth most common malignancy among women, CC has contributed an estimated 660,000 new cases and 350,000 deaths in the year 2022 (1). The impact of the disease is particularly distressing in low- and middle-income countries (LMICs), where inadequate access to human papillomavirus (HPV) vaccination and limited implementation of effective screening programs contribute to persistent health disparities. Epidemiological projections suggest that in the absence of significant preventive and intervention strategies, the incidence and mortality of CC could rise by approximately 56.8% and 80.7%, respectively, by the year 2050 (2). More than 99% of CC cases are causally linked to persistent infection with high-risk HPV genotypes, most notably HPV16 and HPV18, underscoring the critical importance of vaccination, early detection, and sustained global efforts in prevention and control (3).

The oncogenic potential of high-risk HPV lies primarily in the expression of viral oncoproteins E6 and E7, which orchestrate cellular transformation through multiple mechanisms beyond the disruption of p53 and retinoblastoma (pRb) tumor suppressor pathways (4). HPV E6 and E7 engage in more complex interactions with host cellular machinery involving epigenetic reprogramming, metabolic rewiring, immune evasion, and the induction of chromosomal instability (5–7). These viral oncoproteins not only disable cell cycle checkpoints but also actively promote DNA damage accumulation, replication stress, and mitotic errors, creating a cellular environment favorable to malignant transformation (8, 9). Among the 15 recognized high-risk oncogenic HPV types, HPV16 and HPV18 emerged as the predominant drivers, collectively responsible for approximately 70% of CC cases worldwide (10). HPV16 has aggressive carcinogenic potential compared to other high-risk types, such as HPV58, as it exhibits enhanced ability to drive malignant transformation through more efficient disruption of cellular tumor suppressor pathways (11). The HPV16 E7 oncoprotein has been shown to degrade the retinoblastoma protein more effectively compared to the HPV58 E7, resulting in enhanced cell proliferation, invasion, and resistance to apoptosis (12). This differential oncogenic potency among HPV sub-types has important implications in understanding disease progression and developing targeted treatment.

The molecular mechanisms underlying HPV-driven carcinogenesis are propelled by E6 and E7 proteins, which orchestrate cellular transformation through the systematic disruption of host cellular pathways. HPV E6 oncoprotein targets the cellular E3 ubiquitin ligase E6AP (E6-associated protein) to p53, resulting in proteasome-mediated p53 degradation and subsequent loss of cell cycle checkpoint control (13). Simultaneously, E6 disrupts multiple cellular processes by interacting with over 400 host proteins, including transcription factors (p300/CBP), DNA repair proteins (XRCC1, MGMT), apoptosis regulators (Bak, FADD), and PDZ domain-containing proteins involved in cell polarity and adhesion (14).

Among the cellular targets dysregulated by HPV oncoproteins, Aurora kinase B (AURKB) has emerged as an important player in HPV-driven carcinogenesis (15, 16). AURKB, encoded by the AURKB gene, serves as the enzymatic core of the chromosomal passenger complex (CPC), a multiprotein assembly comprising AURKB, survivin (BIRC5), inner centromere protein (INCENP), and borealin (CDCA8) (17, 18). This complex induces critical mitotic processes, including chromosome condensation, kinetochore assembly, spindle checkpoint function, and cytokinesis completion (19). Under physiological conditions, AURKB ensures genomic stability by correcting erroneous kinetochore-microtubule attachments and preventing premature anaphase onset until all chromosomes are properly aligned (20). Studies have revealed that AURKB is frequently overexpressed across multiple cancer types, with particularly elevated levels in CCs (21–23). Aurora A (AURKA) is also upregulated in 60-80% of CCs, correlating with increased tumor aggressiveness and poor prognosis. Moreover, emerging evidence indicates direct mechanistic interactions between HPV E6 oncoprotein and AURKB, involving physical binding that modulates AURKB kinase activity and subcellular localization (15, 23–25). These interactions appear to enhance the oncogenic potential of both viral and cellular components, creating a synergistic effect that accelerates genomic instability and tumor progression.

In CC specifically, AURKA overexpression was identified as an independent adverse risk factor for both recurrence-free survival and overall survival in cervical squamous cell carcinoma patients treated with definitive radical radiotherapy (26). However, AURKB overexpression also serves as a biomarker across multiple malignancies, with significant correlations between elevated expression and reduced survival outcomes established through meta-analytical approaches (27)). These findings from multiple independent cohorts have positioned AURKB not only as a validated prognostic biomarker for risk stratification but also a promising therapeutic target with demonstrated clinical relevance across diverse cancer types (16, 28–30).

The mechanistic basis for enhanced AURKB inhibitor sensitivity in HPV-driven cancers relates to the loss of p53 and pRb function mediated by viral E6 and E7 oncoproteins (15). AURKB inhibition in cells with defective p53 and pRb pathways leads to hyperpolyploidy and subsequent mitotic catastrophe, while normal cells with intact tumor suppressor function can undergo senescence and survive treatment (31). This differential response could provide a therapeutic aspect for the selective targeting of HPV-transformed cells.

This review discusses current understanding of the mechanistic interplay between HPV oncoproteins E6 and E7 and AURKB in cervical carcinogenesis, evaluates the biomarker potential of AURKB for disease prognosis and therapeutic response prediction. We also assessed the therapeutic promise of AURKB inhibition in both monotherapy and combination settings. We examined recent advances in drug development, including novel formulations and delivery approaches, while addressing the challenges and opportunities for integrating AURKB-targeted therapies into precision oncology. Finally, the future research directions and clinical development strategies were presented to transform the management of HPV-driven CC. We critically analyzed the application of biomarker-guided therapeutic interventions which target viral oncogenesis and cellular mitotic dysregulation in CC.

## 2 HPV lifecycle overview and persistence

**Figure 1:**
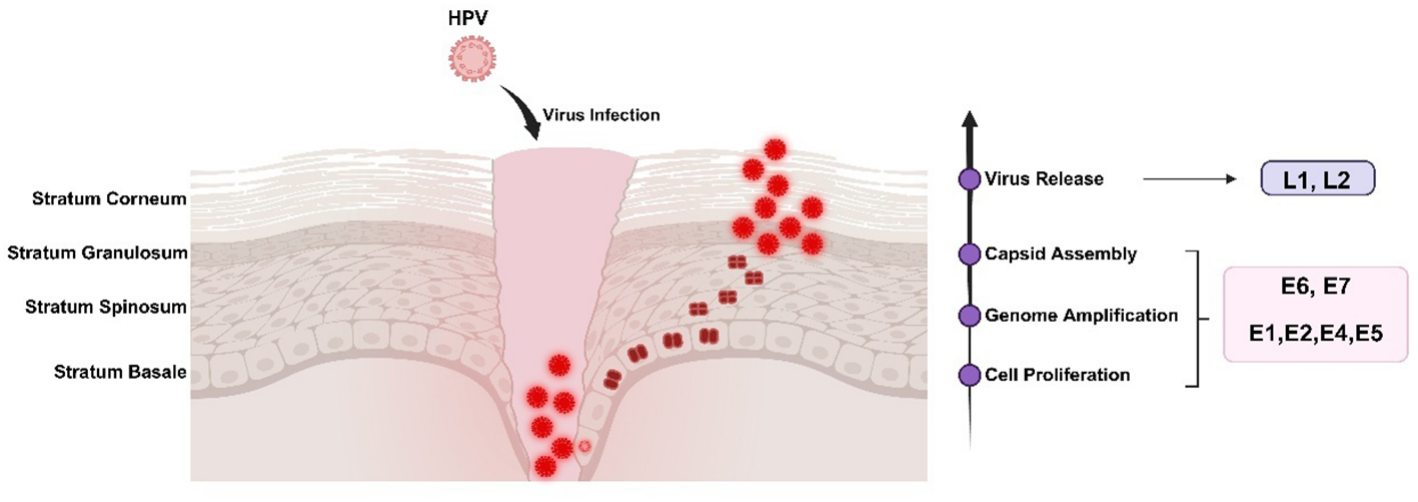
Schematic representation of the Lifecycle of human papillomavirus (HPV) in stratified squamous epithelium. Schematic representation of the Human Papillomavirus (HPV) life cycle within the stratified squamous epithelium. HPV gains entry through microabrasions and infects basal keratinocytes in the stratum basale. Early viral genes (E1, E2, E4, and E5) are expressed to support viral genome maintenance, amplification, and host cell proliferation, as infected cells migrate through the stratum spinosum and stratum granulosum. Oncoproteins E6 and E7 drive cell cycle dysregulation, facilitating viral replication in differentiating keratinocytes. In the upper epithelial layers, late genes L1 and L2 are expressed, leading to capsid assembly. Mature virions are subsequently released from the stratum corneum without cell lysis, completing the viral life cycle.

HPVs are small, double-stranded DNA viruses that specifically target the stratified squamous epithelium (10). The HPV life cycle is connected to cellular differentiation within the host epithelium. Infection typically occurs when micro-abrasions or wounds expose the basal layer of the cervical epithelium, allowing HPV virions to access and bind to basal keratinocytes via heparan sulfate proteoglycans (HSPGs) and the α6/β4 integrin complex (32). After entering the basal cell, HPV reveals its genome, which is transported to the nucleus and persists as an episome. Viral replication proceeds in concert with the host cell cycle, maintaining a low copy number in basal and parabasal cells, which are the cells with proliferative potential and longevity. E1 and E2 viral proteins participate in the initial replication and maintenance of the viral episome (**Figure 1**). The gene expression is tightly regulated at this stage, with very limited expression of the potent viral oncogenes E6 and E7, to avoid triggering host immune detection or premature cell cycle disruption (33).

As the infected basal cells differentiate and migrate upwards through the epithelial layers, a differentiation-triggered regulatory program results in the upregulation of viral gene expression and genome amplification. The E6 and E7 oncoproteins are abundantly expressed, uncoupling the normal cell cycle controls, thus allowing viral DNA to replicate in suprabasal layers where the host cells would otherwise exit the cell cycle and undergo maturation (4). Late viral proteins L1 and L2 are expressed in the uppermost layers, supporting virion assembly and release as the terminally differentiated keratinocytes are shed from the epithelial surface (34). This interaction between the HPV life cycle and epithelial cell differentiation not only facilitates infection but also enables the virus to evade immune surveillance and avoid cell death thereby, contributing to viral persistence and pathogenicity.

Most HPV infections are transient, with the majority of individuals eliminating the virus within 12 to 18 months as a result of humoral and cell-mediated immune responses (35). However, in a subset of individuals (estimated at approximately 10–20%), the infection persists and can be detected on clinical evaluations over extended periods. Persistence of high-risk human papillomavirus (HR-HPV) infection is now recognized as an important determinant of cervical carcinogenesis. Sustained infection with oncogenic HPV genotypes, HPV16 and HPV18, enhances the risk of developing high-grade cervical intraepithelial neoplasia (CIN), which constitutes a critical precursor stage in the progression toward invasive cervical carcinoma (36).

The long-term survival of HPV within host cells is strongly facilitated by the immune-evasive properties of the E6 and E7 oncoproteins, which enable the virus to elude both innate and adaptive immune detection (37). Prolonged infection frequently disrupts the viral E2 regulatory gene, leading to constitutive, unregulated expression of E6 and E7 and thus potentiating cellular transformation (38). Persistent HPV infection is further characterized by the induction of centrosomal and chromosomal instability, both of which are linked to aberrant activities of viral oncoproteins and the deregulation of host genomic integrity. The effect of these events induces epigenetic and genetic alterations that accumulate within infected cells, leading to oncogenic progression (39).

The oncogenic progression driven by HPV is influenced by E6 and E7 proteins. E6 targets the tumor suppressor p53 for proteasomal degradation via the E6-associated protein ubiquitin ligase complex (40). The loss of p53’s regulatory influence abrogates normal cell cycle arrest and impedes apoptosis, thereby facilitating the survival of cells with DNA damage or deregulated proliferative signals. E7 binds to and functionally inactivates the retinoblastoma protein, releasing E2F transcription factors, which are then able to promote S-phase entry and stimulate cellular proliferation independent of normal regulatory checkpoints (41). Together, these interactions result in uncontrolled cell cycle progression, resistance to apoptosis, and accumulation of genomic lesions.

Beyond apoptosis resistance mediated by HPV oncoproteins, disturbances in additional cell death pathways including pyroptosis, necroptosis, and PANoptosis have recently emerged as potentially important contributors to HPV-associated tumor biology. Pyroptosis represents an inflammatory programmed cell death mechanism driven by inflammasome activation and gasdermin-mediated membrane permeabilization, with emerging evidence suggesting roles in CC progression, immune microenvironment modulation, and therapeutic responsiveness (42). Necroptosis, a programmed lytic cell death pathway regulated through receptor-interacting protein kinases RIPK1/RIPK3 and mixed-lineage kinase domain-like protein (MLKL), has also been implicated in tumor progression and immune regulation, thereby potentially contributing to treatment adaptation and therapeutic resistance (43). More recently, PANoptosis, an integrated cell death process incorporating molecular features of pyroptosis, apoptosis, and necroptosis, has been reported. Although direct evidence within HPV-driven CC remains limited, emerging findings suggest that coordinated regulation of these cell death pathways may also influence disease progression and treatment responsiveness (44).

Beyond these canonical mechanisms, the viral oncoproteins E6 and, to a lesser extent, E7 further contribute to the neoplastic transformation of infected epithelial cells by disrupting cell polarity and intercellular adhesion. The E6 protein possesses a C-terminal PDZ-binding motif that enables direct interaction with several PDZ-domain containing scaffold proteins, including DLG1, SCRIB, and MAGI-1, which are essential for maintaining polarity and epithelial junctional integrity (45). Upon binding, E6 recruits the E6-associated ubiquitin ligase E6AP (UBE3A) to mediate the ubiquitin-dependent degradation of these polarity regulators, thereby dismantling the tight and adherens junction complexes that preserve epithelial structure (46). The loss of PDZ-domain proteins destabilizes epithelial organization and disrupts key signaling pathways such as Hippo/YAP, Wnt/β-catenin, and cell surveillance cascades, leading to the loss of contact inhibition, enhanced proliferative and migratory potential, and increased cellular plasticity (47).

Collectively, these molecular perturbations liberate infected cells from normal tissue and regulatory control, enabling an environment conducive to the accumulation of genetic and epigenetic aberrations that drive malignant progression. These viral proteins also drive centrosomal duplication errors and abrogate spindle checkpoint mechanisms, leading to chromosomal instability in high-grade cervical intraepithelial lesions and invasive cancers (48). The persistent activity of HR-HPV oncoproteins is essential not only for tumor initiation but also for the maintenance and malignant progression of cervical neoplasia.

To date, more than 200 HPV genotypes have been identified, among which a subset is classified as high-risk (HR-HPV) because of their strong oncogenic potential, whereas low-risk HPV types are primarily associated with benign proliferative lesions. Persistent infection with HR-HPV types, particularly HPV16 and HPV18, represents the principal etiologic factor underlying cervical carcinogenesis and additionally contributes to several anogenital and oropharyngeal malignancies. However, HPV genotypes differ considerably with respect to tissue tropism, geographic prevalence, oncogenic potential, and associated disease manifestations. Therefore, an overview of major HPV variants and their clinicopathological associations is provided in **Table 1**.

**Table 1:** Major HPV genotypes, anatomical tropism, associated malignancies, and representative HPV-associated diseases.

HPV genotype	Risk category	Primary infection site	Major cancer association	Secondary disease association	Ref
HPV16	High-risk	Cervix, anus, oropharynx, vulva, penis	Cervical cancer, anal cancer, oropharyngeal SCC, vulvar cancer, penile cancer	CIN, VIN, AIN	(49)
HPV18	High-risk	Cervix	Cervical adenocarcinoma	CIN	
HPV31	High-risk	Cervix	Cervical cancer	CIN	
HPV33	High-risk	Cervix, anus	Cervical cancer	Cervical dysplasia	
HPV45	High-risk	Cervix	Cervical adenocarcinoma	CIN	
HPV52	High-risk	Cervix	Cervical cancer	Persistent HPV infection	
HPV58	High-risk	Cervix	Cervical cancer	CIN	
HPV35	High-risk	Cervix	Cervical carcinoma	Cervical dysplasia	
HPV39	High-risk	Cervix	Cervical carcinoma	CIN	
HPV51	High-risk	Cervix	Cervical cancer	Persistent infection	
HPV56	High-risk	Cervix	Cervical cancer	Cervical dysplasia	
HPV59	High-risk	Cervix	Cervical cancer	CIN	
HPV66	Probable high-risk	Cervix	Possible cervical cancer association	Persistent infection	
HPV6	Low-risk	Anogenital epithelium	Rare malignant potential	Genital warts	(50)
HPV11	Low-risk	Anogenital tract, respiratory tract	Rare malignant potential	Genital warts, recurrent respiratory papillomatosis	

## 3 Aurora kinases in CCs

Aurora kinases constitute a highly conserved subfamily of serine/threonine kinases that play indispensable roles in mitosis through the regulation of chromosome segregation, spindle assembly, and cytokinesis (51). AURKA, AURKB, and Aurora Kinase C (AURKC) and their evolutionary conservation and non-overlapping localizations in mitosis underscore their specialized cellular functions, while aberrations in their regulation influence malignancy and CC progression (52). In physiological conditions, these kinases help in accurate chromosome segregation, prevent premature chromosome condensation, ensure appropriate cell division, and contribute to genomic stability (52). Disruption of their expression, structure, or regulation by gene amplification, overexpression, mutation, or epigenetic mechanisms have been closely associated with oncogenesis, aneuploidy, and cervical intraepithelial neoplasia, with findings reported in cervical-, breast-, gastrointestinal-, prostate-, ovarian-, and hematologic cancers (51, 53).

AURKA localizes to centrosomes and spindle poles from late G2 through mitosis and initiates centrosome maturation, separation, and bipolar spindle formation (54, 55). AURKA activation, regulated by phosphorylation at threonine 288, supports mitotic entry and controls the activity of substrates such as targeting protein for Xk1p2 (Targeting Protein for Xklp2- TPX2), nuclear distribution element-1 like-1 (NDEL1), and Eg5 (also known as Kinesin-5) (56). Overexpression of AURKA has been identified in aggressive cervical squamous cell carcinomas and correlates with chromosomal amplification at 20q13.2, resulting in enhanced proliferation, resistance to apoptosis, and transformation (22). Immunohistochemical analyses reveal AURKA overexpression in approximately 50% of squamous CCs, a pattern also observed in breast-, ovarian-, and colorectal malignancies (57). AURKA amplifies oncogenic phenotypes by inhibiting p53, activating nuclear factor kappa beta (NF-κB), and modulating the Wnt/β-catenin pathway, thereby promoting G2/M transition, proliferation, and metastatic potential (26).

AURKB, a serine/threonine kinase regulating chromosomal segregation, is seen predominantly at centromeres and kinetochores during metaphase before transferring to the spindle midzone and midbody in anaphase and cytokinesis (58). AURKB regulates kinetochore-microtubule attachments, spindle checkpoint fidelity, and cell division (51). AURKB’s enzymatic activity, marked by phosphorylation of histone H3 at serine 10 and 28, is indispensable for chromosome condensation and segregation (59). Elevated expression of AURKB is a frequent and early event in the pathogenesis of cervical neoplasms. It has been detected both in CIN and invasive squamous cell carcinoma, with overexpression correlating with CIN grade, tumor stage, and lymph node involvement (57). Xu et al., 2023 developed an AURKB-responsive peptide hydrogel (Gel S/AZD) for controlled delivery of the AURKB inhibitor AZD1152-HQPA to treat CC. The hydrogel induces phosphorylation by activated AURKB, triggering sustained release of AZD and localized inhibition of AURKB signaling. This resulted in reduced phospho-histone H3 expression, cell cycle arrest, and apoptosis in CC cells, with enhanced tumor suppression *in vivo* compared to free AZD (60). AURKB could be used as a marker of high-grade disease but also drives therapeutic resistance by facilitating mitotic arrest and survival following DNA damage, leading to recurrence and disease progression (59).

AURKC, while predominantly expressed in testes and initially linked to meiotic division, is also implicated in CC but only in a few studies, particularly where gene amplifications or aberrant expression have been detected (61). Its functions partially overlap with AURKB, and redundancy might explain the partial compensation observed in certain cancer cell models (62).

All the Aurora kinases share a conserved structural architecture consisting of an N-terminal β-stranded lobe, a glycine-rich region (essential for ATP binding), and a C-terminal α-helical lobe that encompasses the activation or T-loop (63). Autophosphorylation-induced conformational changes within the T-loop are important for attaining full catalytic activity (64). The functional regulation of these kinases is defined by specific interacting partners such as TPX2 for AURKA and INCENP for AURKB, which dictate their subcellular localization, substrate selectivity, and temporal activation during mitosis (53). Alterations or mutations within these structural or regulatory domains result in defective spindle assembly, chromosomal mis-segregation, and polyploidy, (phenotypes frequently detected in CC tissues), thereby establishing the oncogenic role of the Aurora kinase family (65).

## 4 Structural variations and post-translational modifications of AURKB

**Figure 2:**
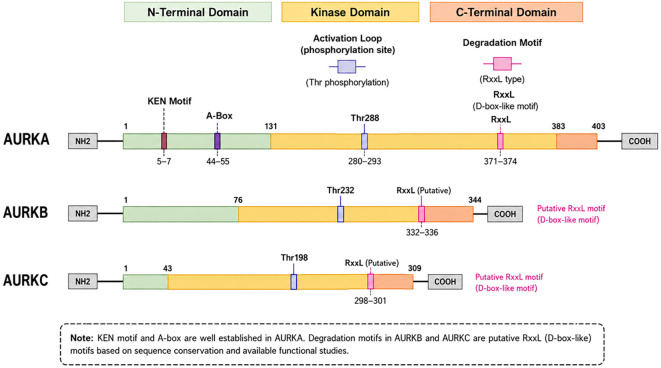
Structural domain organization and key regulatory motifs of AURKA, AURKB, and AURKC. Schematic representation of the domain architecture of Aurora kinase family members showing the N-terminal regulatory domain, catalytic kinase domain, and C-terminal domain. Experimentally reported or predicted regulatory motifs, including KEN motifs, A-box regions, activation loop phosphorylation sites (Thr288 in AURKA, Thr232 in AURKB, and Thr198 in AURKC), and degradation-associated motifs are indicated. Amino acid positions corresponding to motif boundaries and phosphorylation sites are shown. The kinase domains are highly conserved among Aurora kinase isoforms, whereas the N-terminal regions display greater structural divergence. Regulatory motifs may differ in conservation and functional significance across isoforms based on currently available structural and functional studies.

Human AURKB is encoded as a 344-amino-acid serine/threonine kinase containing an N-terminal regulatory domain, a conserved catalytic kinase domain, and a short C-terminal tail essential for Chromosomal Passenger Complex (CPC) interactions (66). Multiple transcript variants of AURKB have been reported. The predominant isoform expressed in proliferating cells retains full kinase activity and CPC-binding capacity, while shorter or truncated variants show reduced stability or altered subcellular localization and are less well characterized (67) (**Figure 2**).

Structural integrity of AURKB is critical for its mitotic functions, particularly its interaction with INCENP, Survivin, and Borealin (68, 69). The binding of INCENP induces a conformational change that stabilizes the activation loop of AURKB, thereby facilitating full catalytic activation (70). Disruption of this structural interaction compromises kinase activity and impairs chromosome alignment and cytokinesis, underscoring the importance of precise structural regulation rather than isoform abundance (71). Post-translational modifications represent the dominant regulatory mechanism controlling AURKB activity, localization, and turnover. Autophosphorylation of AURKB at Thr232 within the activation loop is essential for kinase activation and CPC functionality. This phosphorylation event is tightly coordinated with INCENP binding and serves as a molecular switch for mitotic progression (72). In addition to phosphorylation, AURKB undergoes ubiquitination, which governs its proteasomal degradation during mitotic exit (73). Dysregulation of ubiquitin-mediated turnover leads to aberrant AURKB accumulation, contributing to chromosomal instability and aneuploidy (74).

Emerging evidence also suggests crosstalk between phosphorylation and ubiquitination pathways, whereby sustained phosphorylation stabilizes AURKB and prolongs its mitotic activity in cancer cells (75). Collectively, these findings indicate that while AURKB isoform diversity is limited, structural integrity and post-translational modifications are central determinants of its oncogenic potential, particularly in malignancies characterized by mitotic checkpoint dysfunction.

Twu et al., 2009 conducted a comprehensive analysis of AURKA and AURKB kinase expression in normal cervical tissue, CIN3, and CC. Authors of this study have revealed an upregulation of both kinases in CIN3 and carcinoma compared to normal cervix, with a significant positive correlation between AURKA and AURKB expression, and higher AURKA overexpression observed in squamous cell carcinoma than in adenocarcinoma (57). These findings suggested that AURKA and AURKB overexpression represented an early molecular event in cervical epithelial transformation, implicating these kinases in the early pathogenesis of cervical dysplasia and malignant progression. Sun et al., 2015, explored the role of AURKA in CC by modulating its expression in cell lines. They found that AURKA overexpression promoted cell proliferation, G1/S transition, anti-apoptosis, and resistance to Taxol, while inhibition with VX-680 enhanced apoptosis and chemosensitivity. Clinical samples showed AURKA overexpression with an inverse correlation to pERK1/2, suggesting that AURKA drives CC progression and chemoresistance and may serve as a potential therapeutic target (76). Martin et al., 2017 evaluated the dual inhibitory effects of Alisertib, a selective Aurora kinase inhibitor, in preclinical models of HPV-driven CC. These investigators have demonstrated that Alisertib effectively inhibited both AURKA and AURKB *in vivo*, and that this dual inhibition induced antitumor efficacy and selectivity in CC models. Therapeutic action of Alisertib depends on mitotic progression and might not synergize with agents inducing a G2 DNA damage checkpoint, highlighting that simultaneous inhibition of AURKA and AURKB is essential for effective control of HPV-driven CC (77). Gabrielli et al., 2015 identified AURKA inhibition as lethal in the context of HPV E7 expression, demonstrating that the AURKA inhibitor Alisertib selectively induced apoptosis in HPV-driven CC cells. This effect was mediated by prolonged mitotic delay, resulting in decreased myeloid cell leukemia-1 (Mcl-1) and increased BCL-2-interacting modulator of cell death (BIM) levels, promoting apoptotic cell death. *In vivo*, Alisertib treatment led to tumor regression in HPV-positive xenografts and transgenic models, indicating that targeting AURKA can provide a potentially therapeutic strategy for HPV-driven CC (78). As listed in **Table 2**, foundation studies have shown that Aurora kinase inhibition in CC models disrupts critical pathways involved in cell proliferation, mitotic fidelity, and resistance to apoptosis.

**Table 2:** List of Aurora kinase inhibitors in preclinical and clinical studies on CC.

AKI/target	Structure	Model/system	Major findings/outcomes	References
SNS-314 (Pan-AKI)	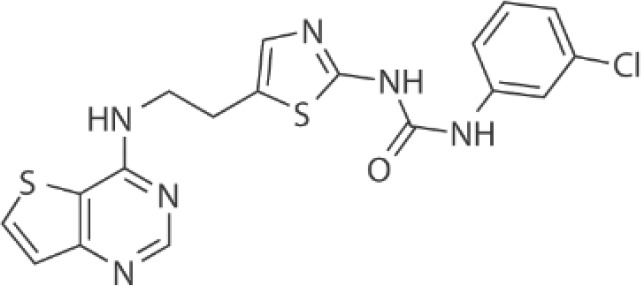	CC Cell lines, xenografts	Additive growth inhibition with chemotherapy; strong antiproliferative effects	(69)
R763/AS703569	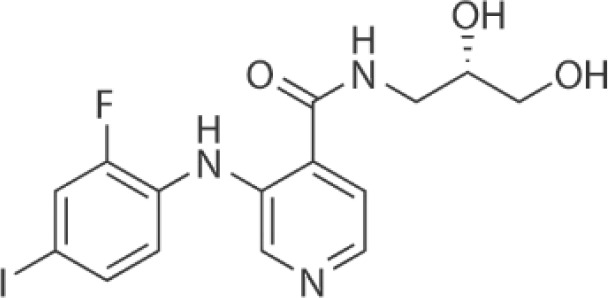	Patient-derived tumor cell lines	Potent Aurora inhibition, broad antitumor activity via mitotic arrest and apoptosis	(70)
Alisertib	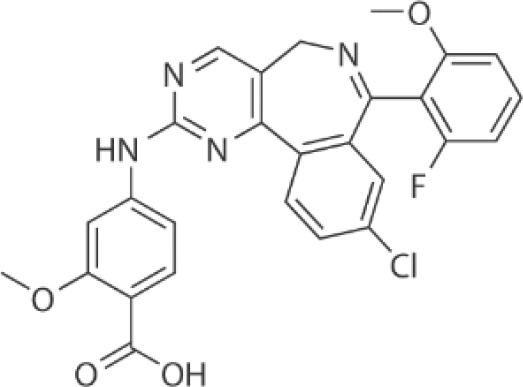	Pdl1–/– CT26/Pdl1–/– MC38 cells	AKI alone upregulates PD-L1, limiting antitumor effects; combination with PD-L1 blockade enhances efficacy	(71)
ENMD-2076 MLN8054 AZD1152 AT9283 Danusertib MK-0457	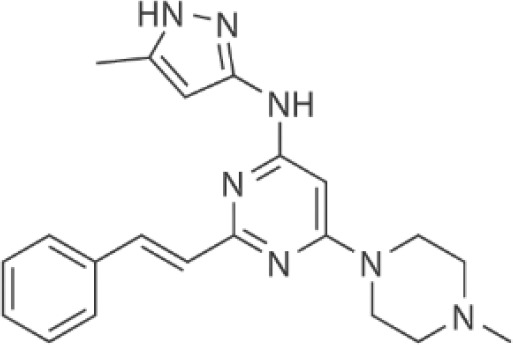 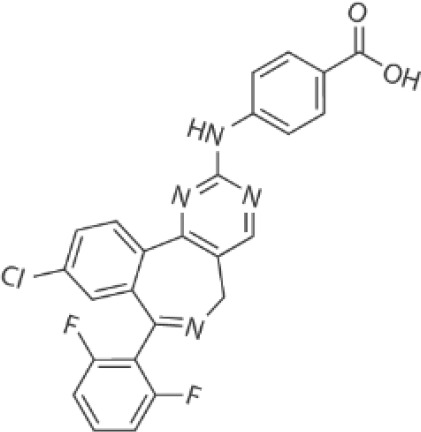 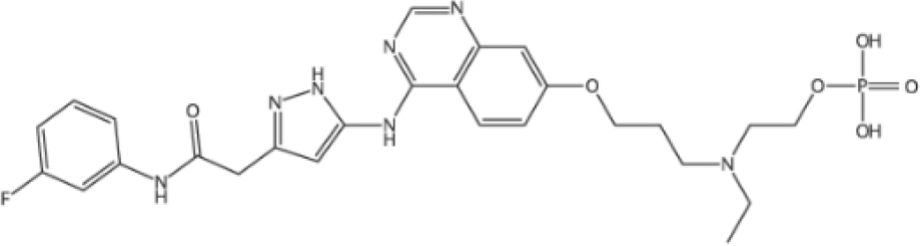 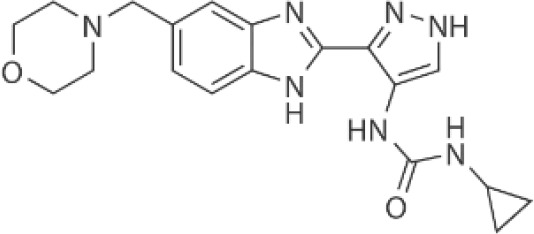 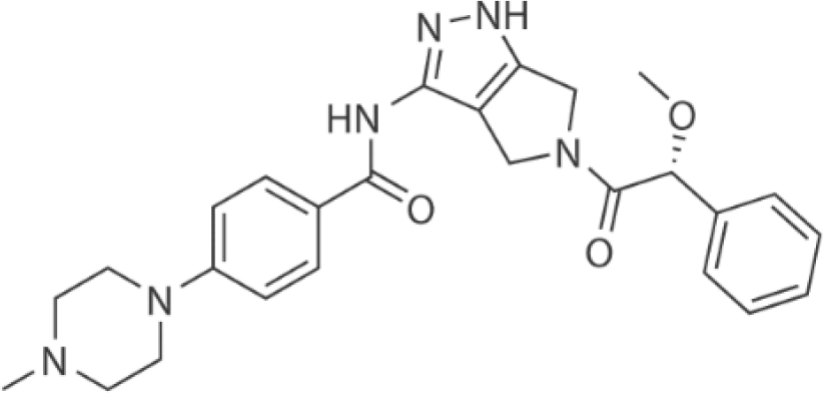 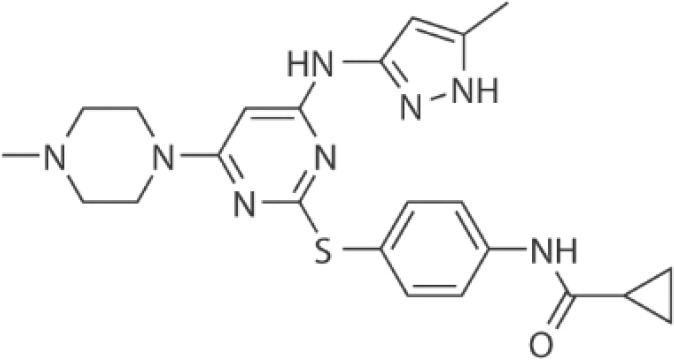	Phase I/II clinical trials	Single-agent and combination activity in gynecologic/solid tumors, partial responses in CC; tolerable toxicity	(72)

Taken together, the Aurora kinase family plays important roles in maintaining chromosomal stability through regulation of spindle assembly and cytokinesis, while its dysregulation, often in concert with HPV oncogenes and checkpoint failures, drives CC pathogenesis. Advancements in next-generation Aurora kinase inhibitors with improved specificity, lower toxicity, and combinatorial potential will highlight their promise as therapeutic agents for enhanced management of cervical malignancies.

### 4.1 HPV and AURKB molecular interplay

Both AURKA and AURKB are frequently overexpressed in HPV-associated malignancies, including CC, with AURKB overexpression observed in approximately 40–60% of HPV-induced tumors (15). This upregulation is correlated with aggressive clinicopathologic features, including advanced tumor stage, increased lymph node metastasis, poor cellular differentiation, and reduced patient survival (16, 79). Mechanistically, E6 and E7 together promote AURKB dysregulation. HPV E6 directly interacts with AURKB through a unique α-helical region located immediately upstream of its PDZ-binding motif, which in high-risk genotypes such as HPV16 and HPV18 corresponds to the C-terminal sequences ETQV and ETQL, respectively (24).

HPV infection induces genomic instability, characterized by elevated phosphorylated AURKB (66). Depletion of E6 and E7 in CC cells resulted in reduced levels of both total and phosphorylated AURKB without significant changes in AURKB transcripts that implied a predominant post-transcriptional mode of viral regulation over kinase function (15). E6 and E7 also modulate the downstream signaling regulated by AURKB by abrogating p53- and pRb-mediated checkpoint control, thereby amplifying mitotic errors, chromosomal instability, and neoplastic transformation (80).

Mechanistically, HPV-mediated regulation of AURKB occur through post-transcriptional and checkpoint-associated mechanisms rather than direct transcriptional activation (15). High-risk HPV E6 physically associates with AURKB through a conserved α-helical domain proximal to its PDZ-binding motif, thereby influence the phosphorylation dynamics of AURKB, its kinase activity, and subcellular localization (81). Studies in HPV-positive cervical cancer cells have demonstrated that depletion of E6 and E7 reduces both total and phosphorylated AURKB protein levels without significantly altering AURKB transcript abundance (15, 24). This suggests that viral oncoproteins regulate AURKB through post-translational mechanisms involving protein stabilization and modulation of kinase activation rather than transcriptional induction.

**Figure 3:**
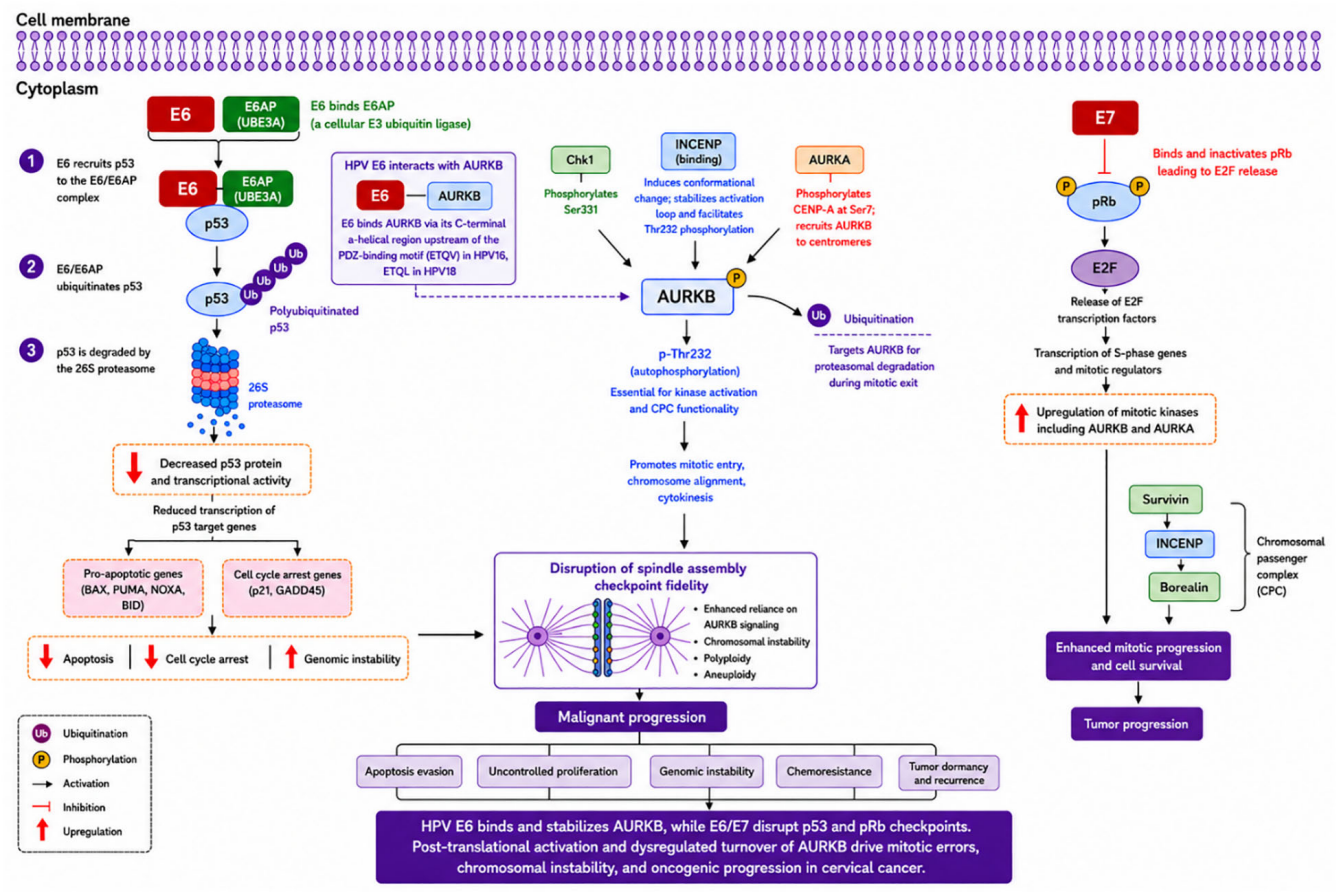
Mechanism of HPV E6/E7-mediated AURKB dysregulation and oncogenic progression in cervical cancer. This figure depicts the role of HPV E6 and E7 oncoproteins in cervical cancer progression through disruption of p53 and pRb tumor suppressor pathways and dysregulation of AURKB signaling. E6 promotes p53 degradation via E6AP-mediated ubiquitination and stabilizes AURKB, leading to abnormal mitotic signaling and chromosomal instability. E7 inactivates pRb, releasing E2F transcription factors and enhancing expression of mitotic regulators including AURKB and AURKA. These combined effects promote genomic instability, uncontrolled proliferation, enhanced cell survival, and malignant progression in cervical cancer.

AURKB catalytic activity is tightly controlled through autophosphorylation of Thr232 residue, which is facilitated by the interaction with INCENP and checkpoint-dependent phosphorylation. These post-translational modifications regulate centromeric localization and chromosomal passenger complex functionality (66). Studies have demonstrated that HPV E6 binding alter AURKB dynamics directly; but the other viral protein ie., E7 contributes indirectly to the AURKB activation by establishing a permissive mitotic environment characterized by checkpoint dysfunction (**Figure 3**) (24).

HPV E6 promotes proteasomal degradation of p53 through E6AP-mediated ubiquitination, while E7 functionally inactivates pRb, releasing E2F transcription factors and overriding G1/S checkpoint control (82). Furthermore, disruption of spindle assembly checkpoint fidelity enhances dependence on AURKB-mediated mitotic signaling, promoting chromosomal instability, polyploidy, and malignant progression (83). Therefore, HPV-driven AURKB activation represents a multifactorial process involving direct viral-host protein interactions, post-translational kinase regulation, and checkpoint deregulation, all of which act synergistically to sustain cervical carcinogenesis.

Transcriptional profiling analyses have revealed upregulation of AURKB in cervical squamous cell carcinoma and endocervical adenocarcinoma versus normal epithelium (16). Recent studies using AURKB inhibitors in both *in vitro* and *in vivo* HPV-positive models have shown the suppression of key oncogenic drivers involved in cell proliferation, telomerase activity, and tumor formation, thereby establishing the therapeutic relevance of the HPV–AURKB interface as a promising target for molecular intervention (24).

Boon et al., 2016 performed a pharmacologic inhibition of AURKB that reduced cell proliferation, induced mitotic defects, diminished telomerase (Human Telomerase Reverse Transcriptase-hTERT) activity in HPV-positive cells, and suppressed tumor formation in xenograft models, which demonstrated that AURKB activity is required for key malignant phenotypes in the HPV context (24). Selective AURKB inhibitors, AZD1152-hQPA induced anti-tumor effects *in vitro* and *in vivo* in HPV-driven models, and AURKB inhibition lowered hTERT protein and telomerase activity in E6-expressing cells (84). The HPV-AURKB interaction is also involved in mechanisms of therapeutic resistance. Elevated AURKB expression has been associated with aggressive clinical behavior as well as reduced sensitivity to DNA-damaging agents in CC cell lines (85). AURKB expression can be induced by platinum agents such as cisplatin, and modulation of AURKB levels alters sensitivity to cisplatin and other chemotherapies in preclinical assays (86).

Another important concern is the potential contribution of AURKB signaling to CC stem cell biology and tumor dormancy, both of which are increasingly recognized as major determinants of recurrence and therapeutic resistance. Cervical CSC populations characterized by stemness-associated phenotypes, including OCT4, SOX2, NANOG, CD44, and ALDH1 expression, possess enhanced tumor-initiating capacity and frequently demonstrate resistance to chemotherapy and radiotherapy, thereby contributing to disease relapse and poor clinical outcomes (87).

Aurora kinase signaling has been implicated in the maintenance of stemness and therapy-resistant cellular states across multiple malignancies. AURKA has been reported to regulate self-renewal pathways involving β-catenin and MYC signaling, while dysregulation of Aurora kinases broadly contributes to genomic plasticity and adaptation to therapeutic stress (88). Although direct evidence specifically linking AURKB to cervical CSC maintenance remains limited, AURKB-mediated regulation of chromosome segregation fidelity, spindle checkpoint control, and mitotic adaptation suggests plausible mechanisms through which persistent AURKB activation may facilitate survival of therapy-tolerant cellular populations (69).

Residual tumor cells surviving initial therapy may enter reversible quiescent states and later contribute to recurrence following prolonged latency periods. Increasing evidence suggests that genomic instability, stress adaptation mechanisms, and checkpoint plasticity influence dormancy-associated therapeutic escape (89). Given the established role of AURKB in chromosomal stability and mitotic checkpoint regulation, determining whether AURKB signaling contributes directly to dormant tumor cell survival or recurrence-associated adaptation remains unanswered.

HPV E6 binds to AURKB via its C-terminal domain, influencing its phosphorylation state and activity. AURKB activation is tightly controlled through phosphorylation at multiple residues, such as Ser331 by Chk1 and Thr232 via INCENP interaction, which regulate its catalytic activity and centromeric localization. Phosphorylation of CENP-A at Ser7 by AURKA facilitates the recruitment of AURKB to centromeres, where this enzyme can further phosphorylate CENP-A at the same site. In HPV-positive CCs, phosphorylated AURKB levels are markedly elevated despite unchanged total protein levels (24). The silencing of E6 and E7 reduces both total and phosphorylated AURKB without affecting AURKB transcript levels, suggesting that HPV-driven AURKB activation is mediated through post-transcriptional mechanisms rather than gene overexpression (90).

Although AURKB overexpression strongly correlates with aggressive clinicopathological characteristics, chromosomal instability, and poor therapeutic outcomes in HPV-associated CC. Current evidence suggests that AURKB primarily functions as a context-dependent oncogenic facilitator rather than an independent initiating driver of cervical carcinogenesis (27). Persistent high-risk HPV infection cause cervical transformation through E6- and E7-mediated disruption of p53 and pRb signaling pathways. In this context, AURKB dysregulation appears to emerge predominantly as a downstream consequence of HPV-mediated cellular reprogramming. However, AURKB contributes functionally to malignant progression by promoting mitotic checkpoint dysregulation, chromosome mis-segregation, cytokinetic failure, telomerase activation, and genomic instability, thereby amplifying oncogenic phenotypes initiated by viral transformation (52, 91, 92). Selective pharmacologic inhibition of AURKB reduces proliferation, impairs tumor growth, diminishes telomerase activity, and induces mitotic catastrophe in HPV-positive models, supporting a major role during tumor maintenance and progression rather than tumor initiation itself (93, 94).

Collectively, these studies show the multiple contributions of AURKB to HPV-mediated cervical oncogenesis. The direct molecular coupling of E6 and E7 to mitotic kinases, as well as the indirect pro-oncogenic consequences of viral disruption of cell cycle control, highlight the importance of AURKB both as a pathogenic effector and as a drug target in HPV-driven malignancies.

### 4.2 Mechanisms, persistence, viral integration, and AURKB in CC progression

Persistent infection with HPV16 and HPV18 is a critical factor in cervical carcinogenesis, facilitating viral genome integration into the host DNA. This integration event disrupts the E2 regulatory gene, which normally represses E6 and E7 oncogenes. Loss of E2 function results in constitutive, deregulated expression of E6 and E7, further promoting oncogenic transformation by targeting tumor suppressors p53 and retinoblastoma protein (pRb) for degradation and inactivation, respectively (95, 96). Integration occurs preferentially at fragile sites within the host genome, including loci such as 3q28 and 8q24, and utilizes DNA damage repair pathways such as microhomology-mediated end joining (MMEJ) (3, 95). This generates genomic instability, chromosomal rearrangements, and epigenetic modifications, ultimately driving neoplastic CC progression.

Integration of HR-HPV DNA into the host genome is important for the transition from precancerous lesions to invasive cervical carcinoma. Multiple large-scale genomic and transcriptomic analyses have shown that the frequency of viral integration increases with lesion severity, being infrequent in low-grade lesions but highly prevalent in high-grade intraepithelial neoplasia and invasive cancers (97, 98). The insertion of HPV DNA frequently disrupts the viral E1 and E2 open reading frames, thereby abrogating transcriptional repression of E6 and E7 (99). The consequent overexpression of E6 and E7 oncoproteins sustains the degradation of p53 and pRb, driving genomic instability and proliferation (100) Jeon et al., 1995 have shown that HPV16 integration stabilizes E6/E7 mRNAs by removing AU-rich elements from the viral early 3′ untranslated region, which normally confer transcript instability (99). Warburton et al., 2018 used high-resolution genomic mapping and showed that HPV integration sites are frequently located near transcriptionally active chromatin domains, enhancers, or fragile regions, leading to virus–host chimeric transcripts, altered chromatin accessibility, and enhancer hijacking (101).

Functional studies demonstrate that selective inhibition of AURKB impaired the survival of HPV-positive CC cell lines and reduced tumor growth *in vivo*. Martin et al., 2017 assessed the relative contributions of AURKA vs AURKB inhibition in HPV-transformed cells, finding a stronger effect with AURKB inhibition in terms of cell viability and mitotic disruption (77). Sheikh et al., 2018 investigated the therapeutic vulnerability of HPV-positive head and neck cancers (HNCs) by targeting the HPV oncogene E7 and Aurora kinases. The study demonstrated that continuous expression of E7 is essential for the survival of HPV-positive HNC cells. RNA interference–mediated silencing of E7 and pharmacological inhibition of Aurora kinases using Alisertib significantly reduced cell viability and tumor growth *in vitro* and *in vivo*. Furthermore, Aurora kinase inhibition led to degradation of the anti-apoptotic protein MCL-1 in E7-expressing cells (102). These findings highlight Aurora kinases as promising therapeutic targets for HPV-driven HNCs, and similar mechanisms could be explored in HPV-associated CC, where E7-mediated oncogenic pathways play a central role. The precise sequence determinants governing E6-AURKB binding, the full range of AURKB substrates in HPV-infected cells, and the interplay between AURKB and other mitotic kinases, AURKA, polo-like kinase (PLK1), and monopolar spindle (MPS) under the influence of viral oncogenes remain poorly understood and have not been extensively investigated (15). While AURKB overexpression correlates with higher-grade cancers and poor outcomes in some HPV-associated tumors, clinical biomarker role of AURKB in CC remains under addressed.

Overall, the molecular interplay between high-risk HPV oncoproteins and AURKB shows how viral infection hijacks host mitotic control to drive cervical carcinogenesis. HPV E6 binds and stabilizes AURKB, enhancing its kinase activity, while E6 and E7 disrupt cell polarity and junctional integrity, creating conditions for aberrant mitosis. The resulting AURKB overactivation promotes chromosomal mis-segregation, aneuploidy, telomerase activation, and genomic instability, ultimately supporting uncontrolled proliferation and malignant transformation. This mechanistic dependency highlights AURKB as a potential therapeutic target in HPV-associated CC, encouraging the development of targeted strategies that integrate viral status and kinase profiling for improved clinical outcomes.

## 5 Aurora kinase dysregulation in cancer

Aurora kinase dysregulation is a recurrent feature of human malignancies, linking defective mitotic control to chromosomal instability, adaptation to genotoxic stress, and therapeutic resistance (66). AURKA and AURKB perform nonredundant, stage-specific roles during mitosis: whereas AURKA governs centrosome maturation, spindle assembly and bipolarity, the AURKB, monitors kinetochore–microtubule attachments, enforces the spindle assembly checkpoint and directs cytokinesis. Disturbances in either node therefore perturbs chromosome segregation fidelity and can drive aneuploidy, tetraploidization and the micronucleation that fuels genome rearrangement and intra-tumoral heterogeneity (103).

Overexpression or hyperactivation of Aurora kinases is observed across a broad spectrum of cancers, including lung, breast, colorectal, ovarian, and cervical carcinomas, where increased AURKA and AURKB expression has been repeatedly documented in pre-neoplastic lesions and invasive tumors (66). Immunohistochemical (IHC) and transcriptomic analyses indicate that upregulation of AURKA is a relatively early event in malignant transformation of the cervical epithelium and is particularly prominent in squamous histology, correlating with higher stage and poorer outcome in several cohorts (57). AURKB overexpression similarly associates with markers of aggressive biology and worse prognosis in multiple tumor types, supporting both kinases as candidate prognostic biomarkers and potential therapeutic targets.

Mechanistically, Aurora kinase overexpression promotes genomic instability through several interlinked processes. Excess AURKA perturbs cytokinesis and promotes centrosome amplification, leading to tetraploidization. Persistent tetraploidy provides a substrate for subsequent chromosomal missegregation and accumulation of structural variants (104). Overactive AURKB alters kinetochore tension sensing and error correction, precipitating lagging chromosomes and micronucleus formation that seed chromothripsis and complex rearrangements. Experimental ablation of AURKB or pharmacologic inhibition produces characteristic phenotypes loss of phosphorylated histone H3 (a proximate AURKB substrate), premature checkpoint silencing, failed cytokinesis, and polyploidy, underscoring the tight link between aurora dysfunction and mitotic catastrophe (105).

Beyond these canonical mitotic roles, studies reveal non-mitotic functions for Aurora kinases that broaden their oncogenic footprint. AURKA and AURKB have been implicated in the regulation of the DNA damage response, transcriptional programs, mitochondrial dynamics, and signaling pathways that control survival and invasion (106). AURKA has been reported to interact with and suppress p53 function, to activate NF-κB and Wnt/β-catenin pathways, and to modulate telomerase activity, thereby facilitating both proliferation and evasion of apoptosis (107). These non-mitotic activities create multiple mechanistic axes through which aurora dysregulation promotes tumor progression, metastatic competence and therapy resistance.

Therapeutic resistance emerges as a predictable consequence of aurora dysregulation. Tumors with elevated aurora activity often display decreased sensitivity to DNA-damaging agents and microtubule poisons because of altered checkpoint dynamics, which permit transient survival despite severe mitotic perturbation (66). Moreover, aurora overexpression can engage survival signaling, including PI3K/AKT and MAPK pathways, and influence expression of anti-apoptotic BCL-2 family members, thereby inhibiting apoptosis and promoting recovery after cytotoxic damages (108). Functional genomic and pharmacologic studies therefore indicate that monotherapy with aurora inhibitors frequently yields cytostatic responses or transient regression, with durable responses limited by adaptive rewiring unless orthogonal vulnerabilities such as such as cell-cycle checkpoint dependence, anti-apoptotic BCL-2 family signaling, PI3K–AKT–mTOR pathway activation, DNA damage response reliance, or spindle assembly checkpoint integrity are simultaneously targeted (109).

The interplay with oncogenic viruses underscores a context in which aurora kinase dysregulation becomes particularly pathogenic. In HPV-driven cancers, viral oncoproteins E6 and E7 disable p53 and Rb checkpoints and have been reported to modulate aurora kinase stability and activity (15). Direct biochemical interactions between E6 and AURKB have been described and linked to increased telomerase activity and immortalization phenotypes, providing a mechanistic basis for how viral infection amplifies aurora-dependent genomic instability and creates selective pressure for aurora dependency in transformed cells (24). This viral-host interaction suggests that aurora inhibition may use a virus-specific vulnerability, but it also complicates the prediction of response because post-translational regulation, rather than transcriptional upregulation, often underlies elevated aurora activity in the HPV context (8).

Genetic screens and RNA interference studies have identified AURKA as a synthetic vulnerability in certain HPV-positive cell lines, and selective AURKA inhibition can produce tumor regressions in animal models of HPV-transformed disease, indicating potential for a selective therapeutic index in virally driven tumors (78). Conversely, pan-aurora inhibitors and less selective agents have been constrained clinically by hematologic toxicity and off-target effects, underscoring the need for more selective chemistry, optimized dosing regimens, and rational combination strategies to translate pathway inhibition into meaningful patient benefit (110). The studies to date, therefore, support pairing AKIs with agents that abrogate compensatory survival, with DNA damage response inhibitors (PARP, CHK1/ATR, or WEE1), or with immunotherapeutic approaches rather than reliance on single-agent activity (111).

Overall, Aurora kinases present both promise and challenges as therapeutic targets. Their essential role in mitosis makes them attractive for inducing mitotic failure in cancer cells. However, because Aurora kinases are also active in normal proliferating tissues, effective treatment strategies must improve the therapeutic window through isoform-selective inhibitors, tumor-targeted delivery, and rational drug combinations that allow lower and less toxic dosing. In addition, since Aurora kinase dysregulation contributes to genomic instability, inhibition may temporarily limit tumor evolution but also promote the emergence of alternative mitotic adaptation mechanisms. Continuous monitoring of tumor clonal dynamics during therapy will therefore be important to detect and manage resistance early. Further progress in targeting Aurora kinases will depend on integrating fundamental mechanistic insights with well-designed translational trials that incorporate functional biomarkers, adaptive treatment strategies, and biologically informed combination therapies. This approach provides a rational path to effectively exploit Aurora kinase dysregulation across multiple cancer types, including cervical carcinoma. The mechanistic basis for these strategies, along with representative examples of Aurora kinase alterations in cancer, is supported by key foundational reviews and primary studies.

### 5.1 Molecular functions and dysregulation of AURKB

AURKB maintain genomic stability in normal cells by regulating chromosome condensation, correcting erroneous kinetochore-microtubule attachments, securing proper chromosome alignment, and activating the spindle assembly checkpoint (SAC) to prevent missegregation (112, 113). Together with its CPC partners, AURKB localizes first to chromosome arms, then centromeres, and finally the central spindle and midbody as mitosis proceeds (114). When chromosomes are not properly attached, AURKB destabilizes these connections by phosphorylating multiple substrates, permitting corrective realignment to facilitate accurate segregation (114). AURKB also plays a vital role in cytokinesis, where the loss or inhibition of AURKB results in failed cytokinesis and promotes the formation of polyploid, genomically unstable cells (115, 116). Dysregulation of AURKB, in HPV-driven malignancies, contributes significantly to tumorigenesis. In CC, AURKB overexpression is observed in 40-60% of HPV-positive tumors, contributing to chromosomal instability and increased tumor aggressiveness (15). HPV E6 oncoprotein can interact directly with AURKB in the nucleus of mitotic cells, promoting E6-AURKB complex formation. This molecular interaction paradoxically reduces AURKB kinase activity but increases hTERT protein and telomerase activity, facilitating cell immortalization (24).

One of the key molecular functions of AURKB is the regulation of chromosome microtubule attachments (117). During prometaphase and metaphase, AURKB localizes to centromeres, where it is ideally positioned to monitor and correct erroneous kinetochore-microtubule attachments (116). AURKB introduces this by phosphorylating various kinetochore and centromere proteins, including Hec1, Dsn1, and the Ndc80 complex, thereby destabilizing improper attachments (118). This phosphorylation activity leads to the establishment of proper amphitelic attachments, where each chromosome is attached to spindle poles from opposite sides (119).

AURKB is also known to be a principal regulator of the spindle assembly checkpoint (SAC). The SAC ensures that cells do not proceed to anaphase until all chromosomes have attained proper alignment at the metaphase plate and stable bipolar attachments (120). AURKB regulates SAC activation both by generating tension at correctly attached kinetochores and by sustaining the accumulation of checkpoint proteins such as BUBR1, MAD2, and Mps1 at unattached kinetochores (119). Inhibition or loss of AURKB leads to rapid checkpoint silencing, premature anaphase onset, chromosomal mis-segregation, and the generation of micronuclei in daughter cells, all of which contribute to genome instability (115, 121).

Another crucial phase of mitosis regulated by AURKB is cytokinesis, where, during anaphase and telophase, together with the rest of the CPC, it translocates to the central spindle (122). AURKB is shown to promote the assembly and constriction of the actomyosin, coordinates the reorganization of the central spindle microtubules, and directly regulates proteins such as MKLP1 and CIT-K (116). Misregulated AURKB activity impairs the cytokinetic abscission process, resulting in the generation of multinucleated or polyploid cells. This mitotic failure induces persistent chromosomal instability, which promotes aggressive tumorigenesis through the accumulation of genetic aberrations (115, 116).

Upregulation of AURKB in HPV-positive cancers is primarily regulated post-transcriptionally and involves direct interactions between AURKB and viral oncoproteins. It has been shown that E6 from high-risk HPV can physically interact with AURKB in the nucleus of mitotic cells, forming a complex with yet unclear molecular effects (24). Although this complex is associated with reduced AURKB kinase activity, it correlates with elevated telomerase (hTERT) and increased cell immortalization, providing a mechanism for the oncogenic synergy between HPV infection and host mitotic kinases (123). HR-HPV types influence the degradation of tumor suppressors such as p53 and pRb via E6 and E7, respectively, further enhancing the tolerance for chromosomal instability introduced by AURKB overexpression or hyperactivation (66).

### 5.2 Mechanisms of resistance to AURKB inhibition and predictive biomarkers

Despite the preclinical activity, resistance to AURKB-targeted therapy remains a major challenge that limits durable therapeutic responses. Similar to other mitotic kinase inhibitors, tumor cells may acquire adaptive mechanisms that bypass mitotic stress and restore proliferative capacity (124). One such mechanism involves activation of parallel survival signaling pathways, including PI3K/AKT/mTOR and MAPK/ERK cascades, which promote cellular survival despite mitotic disruption induced by AURKB inhibition (67). Upregulation of anti-apoptotic proteins such as BCL-2, BCL-XL, and MCL-1 may further reduce treatment-induced apoptosis, contributing to resistance development (125).

Alterations in cell-cycle checkpoint regulators are shown to influence sensitivity to AURKB inhibitors. It is known that small-cell lung cancer cells harboring defective p53 or pRb pathways frequently exhibit increased dependency on mitotic regulators and enhanced susceptibility to AURKB inhibition (126). Given that CC commonly exhibits dysregulation of p53 and pRb signaling, particularly in HPV-associated disease, a similar vulnerability to AURKB inhibition may potentially exist in CC cells. However, prolonged therapeutic exposure can promote adaptive rewiring of spindle assembly checkpoint (SAC) signaling, DNA damage response pathways involving ATR, CHK1, and WEE1, and compensatory mitotic kinase activity mediated by AURKA, polo-like kinase 1 (PLK1), and monopolar spindle kinase 1 (MPS1), thereby restoring mitotic progression and cellular survival. Functional redundancy among mitotic kinases represents an important mechanism of resistance in tumors exposed to prolonged Aurora kinase inhibition.

Emerging evidence further suggests that genomic and molecular biomarkers may improve patient stratification for AURKB-targeted therapy. Elevated AURKB expression, HPV-positive molecular status, phospho-AURKB abundance, spindle checkpoint dependency, and defects in p53 and pRb signaling may predict improved therapeutic responsiveness. In contrast, activation of PI3K/AKT signaling, elevated anti-apoptotic protein expression, and enhanced DNA damage repair capacity may identify tumors less likely to respond to monotherapy approaches. These observations support rational combination strategies incorporating AURKB inhibitors together with DNA damage response inhibitors, immune checkpoint blockade, PI3K pathway inhibitors, or anti-apoptotic targeting approaches to overcome resistance mechanisms and improve therapeutic durability.

Despite preclinical evidence supporting AURKB inhibition in HPV-driven malignancies, important translational challenges remain before these strategies can be broadly implemented in clinical practice. Although AURKB inhibition demonstrates potent anti-proliferative activity and mitotic disruption, translating these effects into clinics remains challenging because Aurora kinases also regulate normal proliferative tissues, thereby narrowing the therapeutic window.

Clinical development of Aurora kinase inhibitors has additionally been limited by hematologic toxicities, dose-limiting neutropenia, gastrointestinal adverse events, and the emergence of adaptive resistance pathways (127, 128). These limitations suggest that biomarker-guided therapeutic approaches may be required to identify patient populations most likely to benefit from treatment. HPV molecular status, phospho-AURKB expression levels, spindle assembly checkpoint dependency, p53 and pRb pathway alterations, and mitotic vulnerability signatures may represent potential predictive biomarkers requiring prospective validation (129).

Monotherapy approaches may provide limited long-term efficacy due to signaling networks and mitotic adaptation mechanisms can restore tumor survival. Combination strategies integrating AURKB inhibitors with chemotherapy, radiotherapy, immune checkpoint blockade, or DNA damage response inhibitors may therefore improve therapeutic durability while reducing selective evolutionary pressure that promotes resistance (66). Future translational advancement will require biomarker-enriched clinical trials, patient-derived experimental systems, and mechanistically informed therapeutic combinations capable of bridging promising preclinical observations toward clinically meaningful benefit in HPV-associated cervical cancer.

### 5.3 Aurora kinase inhibition as a novel therapeutic strategy for CC treatment

Numerous Aurora kinase inhibitors (AKIs) have been developed to suppress the oncogenic signaling of Aurora kinases, with several compounds advancing into preclinical and clinical evaluation (130, 131). AKIs are broadly classified into (a) pan-Aurora inhibitors, which act on all three family members due to structural conservation, and (b) isoform-selective inhibitors, which preferentially target either AURKA or AURKB. Representative pan-inhibitors include MK-0457 (132), BI-847325 (133), PHA-739358 (27), and ENMD-2076 (134), while MLN8054, MLN8237 (Alisertib), and MK5108 (VX-689) selectively inhibit AURKA, and BI-811283 and AZD2811 target AURKB (135, 136).

Pan-inhibitors generally demonstrate broader antitumor efficacy but are associated with higher systemic toxicity, whereas selective inhibitors offer improved tolerability but carry a higher risk of resistance development. Among specific inhibitors, Alisertib, which is AURKA -specific, and AZD2811, which targets AURKB, have reached phase III clinical trials for advanced solid tumors (78). Functional genomic screening using siRNA identified AURKA as an essential survival factor in HPV-positive CC cells, and sensitivity to Alisertib was shown to correlate with HPV E7 expression levels (78). Other promising AKIs such as PHA-739358, which inhibits AURKA/B along with kinases including FGFR, Abl, and RET, and AT9283, a multitarget inhibitor of Aurora kinases, JAK2/3, and Abl, also display broad-spectrum antitumor potential (137). Although AURKA and AURKB overexpression are important events in CC, inhibition of these kinases alone has yielded limited therapeutic benefit (138). However, combination regimens have shown substantial improvements in efficacy across diverse tumor types, including HPV-associated cancers. Co-targeting Aurora kinases with inhibitors of anti-apoptotic proteins such as BCL-2 (Venetoclax), BCL-XL (A1331852), or Mcl-1 (A1210477) markedly enhanced cell death in CC models, possibly through prolonged mitotic arrest and checkpoint disruption (139). In Rb-deficient, HPV-positive cells, overexpression of the mitotic checkpoint protein MAD2 (MAD2L1) drives dependency on both TRIP13 and Aurora kinases for mitotic fidelity. Simultaneous inhibition of TRIP13 and Aurora activity with Alisertib induced irreversible mitotic arrest, apoptosis, and DNA damage in head and neck squamous cell carcinoma (HNSCC) and CC cell lines (140). Similarly, dual inhibition of AURKA and AURKB with PF-03814735 triggered compensatory activation of the EGFR–ERK signaling axis; blocking this pathway further potentiated the drug’s antitumor efficacy in patient-derived CC organoids (138).

Aberrant activation of the PI3K/AKT/mTOR has been frequently observed in cervical and other gynecologic malignancies (141–143). Pharmacological inhibition of PI3K using Alpelisib has shown promise in combination therapies (144–146). In CC models, concurrent inhibition of PI3K and AURKA with Alpelisib and Alisertib produced synergistic cytotoxic effects, characterized by enhanced apoptosis, increased PARP cleavage, prolonged mitosis, and failed cytokinesis (147). These findings suggest that dual blockade of PI3K and AURKA signaling enhances chemosensitivity and overcomes intrinsic resistance mechanisms.

The ATP13A3 transporter, a P-type ATPase involved in ion transport, has been identified as a negative prognostic marker in HNSCC and high-grade cervical lesions (148). Elevated ATP13A3 expression was correlated with increased AURKA activity and poor clinical outcomes. Silencing ATP13A3 reduced AURKA protein levels, suggesting that patients with high ATP13A3 expression may benefit from AURKA -targeted therapies (149).

AURKB plays a pivotal role in chromosomal segregation and cytokinesis, while kinesin spindle protein (KSP) is essential for bipolar spindle formation (150, 151). Inhibition of either protein alone produces limited efficacy due to compensatory signaling through cyclin B degradation and apoptotic regulators (152). Nonetheless, combining AURKB or KSP inhibitors with drugs targeting anti-apoptotic proteins enhances therapeutic outcomes. Dual inhibition of BCL-XL with Navitoclax and either Ispinesib or Barasertib (AURKB inhibitor) significantly augmented mitotic cell death in oral squamous carcinoma cells (152, 153).

In HNSCC, NOTCH1 truncating and missense mutations are common, and PI3K inhibition selectively targets NOTCH1-mutant tumors (154). Synergistic suppression of AURKB and PI3K led to enhanced apoptosis and durable tumor regression *in vivo*. Mechanistic studies revealed that AURKB overexpression upregulates AKT1 and PDK1, supporting NOTCH1-mutant tumor survival under PI3K blockade. Thus, dual inhibition of PI3K and AURKB defines a promising strategy for treating NOTCH1-mutant HNSCC (154).

Genome-scale RNA interference screens in HNSCC models have also identified AURKA as a critical survival kinase, where its depletion induces apoptosis and loss of viability across multiple cell lines (155, 156). Aurora kinases, along with PLK1, WEE1, and TTK, were also implicated in TNFα-induced NF-κB activation and G2/M checkpoint regulation, revealing new druggable vulnerabilities (157). A recent clinical investigation combining Alisertib and Pembrolizumab in advanced HNSCC demonstrated that AURKA inhibition can overcome immunotherapy resistance in Rb-deficient tumors, achieving prolonged disease stabilization in previously refractory patients (158). In summary, Aurora kinase inhibition, particularly in combination with targeted or immune-based therapies, offers a versatile and promising therapeutic avenue for HPV-related CC.

### 5.4 Crosstalk with HPV oncogenic pathways and AURKB

Mechanistic studies have demonstrated that AURKB is regulated predominantly at the post-translational level in HPV-driven malignancies, with the HPV E6 and E7 oncoproteins modulating AURKB protein stability and phosphorylation rather than its mRNA expression (15). Depletion of HPV E6 and E7 leads to marked reductions in both total and phosphorylated AURKB protein without significant changes at the RNA level, highlighting a layer of viral-mediated post-translational regulation (24). E6 is known to trigger ubiquitin-mediated destruction of p53, thereby disabling the DNA damage response, while E7 binds and inactivates Rb, liberating E2F target genes and promoting G1/S cell cycle transition (159, 160). The effect is a loss of mitotic checkpoint control, a condition that greatly amplifies the consequences of AURKB overexpression, as cells become increasingly tolerant of chromosome missegregation and polyploidy (121).

Cell proliferation in HPV-driven cancers is due to hyperactivation and stabilization of AURK, and also deactivation of p53 and Rb. This molecular environment accelerates the cell cycle, limits apoptosis, and undermines the genomic stability required for normal tissue homeostasis (102). AURKB is known to phosphorylate p53 at Ser315, marking it for proteasomal degradation and further suppressing pro-apoptotic genes such as BAX and BAD (66). Additionally, AURKB supports STAT3 phosphorylation at Ser727, thereby enhancing transcription of anti-apoptotic genes and increasing cancer cell survival (66).

The pro-metastatic potential of AURKB is also underscored by its role in epithelial-mesenchymal transition (EMT) and extracellular matrix remodeling. Overexpressed AURKB upregulates the activity of matrix metalloproteinases (MMPs), enzymes that enable cancer cells to degrade surrounding tissue and invade distant organs (116). Activation of focal adhesion kinase (FAK) and Rho GTPase pathways driven by AURKB further enhances cell migration and motility, promoting metastatic progression (66). In CC models and other HPV-related tumors, AURKB silencing or inhibition results in potent anti-proliferative effects, restoration of apoptotic pathways, impaired EMT, reduced motility, and loss of metastatic potential (27).

Collectively, these findings establish that AURKB acts as a central molecule for crosstalk with HPV oncogenic pathways in CC. The synergistic disruption of cell cycle checkpoints, mitotic surveillance, and apoptotic programs by the combined action of HPV oncoproteins and AURKB overexpression accelerates malignant transformation, supports cell survival under stress, and enables dissemination.

## 6 Combination therapies

Combination therapies involving aurora kinase inhibitors represent a rapidly evolving strategy in the management of CC, particularly for tumors associated with HPV infection (161). The therapeutic targeting of AURKA and AURKB has thus emerged as a rational approach, with growing preclinical and clinical evidence supporting the enhanced efficacy of combination regimens over monotherapy. Preclinical investigations have demonstrated that aurora kinase inhibitors, such as MLN8237 (Alisertib) and AZD1152-hQPA (Barasertib), exhibit potent anti-proliferative effects in CC cell lines (139). Zhang et al., 2011 investigated the role of AURKB in CC and the effects of its inhibition with ZM447439, alone and in combination with Cisplatin. The study found that AURKB was highly expressed in CC tissues and that its inhibition suppressed cell growth and induced apoptosis. Combined treatment with ZM447439 and Cisplatin produced a stronger inhibitory and apoptotic effect than either drug alone, suggesting a synergistic interaction. The dual treatment also reduced the expression of oncogenic and anti-apoptotic proteins while increasing P53 levels. Overall, the findings indicate that targeting AURKB may enhance the sensitivity of CC cells to chemotherapy and serve as a potential therapeutic approach (23). Sootome et al., 2020 demonstrated that TAS-119, a novel oral and selective AURKA inhibitor, enhanced the antitumor efficacy of taxanes such as paclitaxel and docetaxel. TAS-119 increased the growth-inhibitory effects of taxanes, including resistant ones, without affecting normal cells. In CC animal models, the combination improved antitumor activity without worsening taxane-associated toxicities like neutropenia or neurotoxicity. These findings highlighted TAS-119 as a promising agent for combination therapy with taxanes (162).

Dual inhibition of aurora kinases and anti-apoptotic BCL-2 family members produced rapid and enhanced cell death in CC cell lines. Alisertib combined with small-molecule antagonists of BCL-2, BCL-XL, or MCL-1, caused apoptosis during mitotic delay. These findings identified that aurora kinase inhibitors-induced mitotic stress created a dependence on anti-apoptotic proteins that could be therapeutically exploited (139).

Inhibition of stress-activated kinases has been demonstrated to potentiate the therapeutic efficacy of aurora kinase inhibition in CC models. Specifically, pharmacologic blockade of p38 MAPK using BIRB796 has been shown to enhance the antitumor activity of the pan-Aurora kinase inhibitor VX-680 (MK-0457) in CC cells. The combined treatment produced tumor growth suppression compared with either agent alone, both *in vitro* and in xenograft models. Mechanistically, activation of the p38 MAPK pathway following mitotic disruption facilitated cellular survival and recovery from mitotic stress. Consequently, concurrent inhibition of p38 MAPK abrogated this adaptive response, thereby amplifying apoptosis and mitotic arrest induced by aurora kinase inhibition. This dual-targeting approach effectively disabled compensatory stress-response signaling, providing a strategy to enhance the cytotoxic potential of aurora kinase inhibitors in CC (163).

Zhang et al., 2011 investigated the role of AURKB in CC and determined how its inhibition with ZM447439 affects SiHa cells, both alone and in combination with cisplatin. They found that AURKB is highly expressed in CC tissues and that blocking its activity reduces cell growth and promotes apoptosis. ZM447439 and cisplatin each suppressed cell proliferation, but their combination produced a much stronger, synergistic effect, leading to enhanced S-phase arrest and early apoptosis. The dual treatment more effectively decreased HPV16E6 and BCL-2 while increasing P53 expression, suggesting a stronger pro-apoptotic response (23). Overall, AURKB was suggested to be a promising therapeutic target in cervical squamous carcinoma and that its inhibition can enhance the chemosensitivity of CC cells to cisplatin.

Jin et al., 2016 examined the effects of the Aurora kinase inhibitor VX-680 on CC cells and explored whether blocking p38 MAPK could enhance its pro-apoptotic activity. They observed that VX-680 inhibited CC cell proliferation by inducing G2/M arrest, but activation of p38 MAPK reduced apoptosis. Inhibiting p38 MAPK with BIRB796 eliminated p-p38 signaling and increased VX680-induced cell death. The combination also showed stronger tumor suppression in a mouse xenograft model (163)). Overall, the study suggested that dual inhibition of Aurora kinases and p38 MAPK produced synergistic antitumor effects and represents a promising therapeutic strategy for CC.

Martin et al., 2017 demonstrated that Alisertib, although primarily considered an AURKA inhibitor, effectively inhibits both AURKA and AURKB in preclinical models of HPV-driven CC. Their findings show that dual inhibition of these kinases is essential for the drug’s selectivity and antitumor efficacy. They also showed that alisertib relies on cells progressing through mitosis to exert its therapeutic effects and is therefore unlikely to interact with agents that enforce a G2 DNA-damage checkpoint (77). Overall, the study highlights that simultaneous targeting of AURKA and AURKB is necessary for effective treatment of HPV-driven cancers using Aurora kinase inhibitors. The principal preclinical combination studies and selected clinical combination trials in CC are provided in **Table 3**.

**Table 3:** List of preclinical and clinical studies targeting Aurora kinases in CC.

Aurora kinase inhibitor	Co-administered agent	Experimental model	Outcome	References
TAS-119	Paclitaxel/docetaxel	HeLa; Taxane-resistant lines; *in vivo* xenografts	TAS-119 enhanced the antiproliferative activity of Paclitaxel, Docetaxel and Cabazitaxel *in vitro* and increased antitumor efficacy *in vivo* without exacerbating taxane toxicities; optimal schedule defined	(162)
MK-5108	Docetaxel	HeLa-luc xenografts and multiple tumor cell lines; *in vivo* models (nude rats/mice)	MK-5108 induced mitotic accumulation in HeLa models and potentiated Docetaxel antitumor activity in xenografts; combination produced greater tumor growth inhibition than Docetaxel alone without markedly increasing toxicity in preclinical studies.	(164)
AZD1152-HQPA	All-trans retinoic acid (ATRA), Am80, TAC-101 (retinoid-class agents)	HeLa and cisplatin-resistant HCP4 cervical cell line	AZD1152-HQPA displayed antagonism with some genotoxics but synergized with retinoid agents (ATRA, Am80, TAC-101) in HeLa cells; outcome suggests interaction between AURKB expression modulation and retinoid-mediated differentiation/apoptosis pathways.	(84)
AMG-900	Microtubule-targeting agents (taxanes)	HeLa; multiple xenograft models	AMG-900 inhibited phosphorylation of histone H3 in HeLa, induced polyploidy and apoptosis, and potentiated activity of microtubule-targeting agents in taxane-resistant models; activity observed at low nanomolar concentrations.	(165)
WEE1	Adavosertib	Although the primary combination study was in HNSCC and other squamous models, these tumors share cell-cycle dependencies with HPV-related CCs; HeLa cells	Combined AURKA and WEE1 blockade increased replication stress, mitotic catastrophe and apoptosis more than either single agent; readouts: replication stress markers (RPA phosphorylation), γ-H2AX, cell cycle aberrations, tumor growth suppression in xenografts—supports testing in cervical models.	(166)

Together, these studies have demonstrated the wide range of promising combination strategies involving aurora kinase inhibitors, and underscore the need for CC focused clinical testing that incorporates biomarker-guided patient selection and optimized dosing schedules. Continued preclinical work on treatment timing, along with translational trials that include correlative analyses, will be essential to fully realize the therapeutic potential of aurora kinase inhibitor–based combinations in CC.

## 7 Challenges in developing Aurora kinase inhibitors for CC

Despite preclinical evidence supporting Aurora kinase (AURK) inhibitors as rational components of combination therapy for CC, multiple challenges continue to limit their successful clinical integration. A central obstacle is the biological heterogeneity of CC, particularly in the context of HPV-driven oncogenesis. Although Aurora kinase dysregulation is closely linked to HPV oncogene-mediated perturbation of cell-cycle checkpoints, centrosome amplification, and chromosomal instability, dependency on AURKA versus AURKB varies substantially across tumors (15). AURKA amplification is observed only in a subset of CC cases, and its prognostic and predictive significance remains incompletely defined, complicating patient selection (166). Similarly, while AURKB overexpression correlates with poor prognosis and aggressive proliferation, not all tumors exhibit functional reliance on AURKB activity, underscoring the necessity of molecular stratification (57).

Therapeutic resistance represents a further major challenge. Both intrinsic and acquired resistance to AURK inhibitors have been documented across cancer types and are likely seen in CC. Resistance frequently emerges through compensatory activation of survival pathways, including PI3K/AKT, MAPK, and spindle assembly checkpoint regulators, which preserve cell viability despite mitotic disruption (131).

Pharmacologic limitations of current AURK inhibitors further restrict their clinical utility. Agents such as Alisertib and Barasertib exhibit narrow therapeutic indices, with dose-limiting hematologic toxicities arising from on-target effects in proliferating normal tissues (167). These toxicities are often worsened when AURK inhibitors are combined with cytotoxic chemotherapy, complicating dose optimization and treatment tolerability. In addition, limited isoform selectivity and overlapping inhibition of AURKA and AURKB contribute to off-target effects (66). Although next-generation inhibitors with improved selectivity have been developed, their efficacy in CC remains largely unexplored. Clinical translation of Aurora kinase inhibitors has also been constrained by pharmacokinetic and therapeutic exposure limitations. Early-generation Aurora kinase inhibitors frequently demonstrated pharmacologic challenges, including dose-limiting toxicities that complicate sustained target inhibition while minimizing systemic adverse effects. Barasertib (AZD1152), despite potent AURKB inhibitory activity, demonstrated significant hematologic toxicities, particularly neutropenia, in phase I solid tumor studies, which limited dose intensity and broader clinical applicability (168). Danusertib (PHA-739358), a pan-Aurora kinase inhibitor, demonstrated manageable pharmacokinetic characteristics of Aurora pathway inhibition, however, dose-limiting neutropenia, gastrointestinal toxicities remained translational barriers in advanced solid tumor studies (169).

These observations emphasize that successful future implementation require biomarker-guided patient selection, optimized pharmacokinetic properties, improved isoform specificity, and rational combination approaches to maximize efficacy while minimizing systemic toxicities. Advanced nanoparticle-based Aurora kinase formulations such as AZD2811 have therefore emerged as promising approaches to improve pharmacokinetic behavior and toxicity efficacy balance in solid tumors (170). Treatment scheduling complexity is another limitation. Preclinical studies demonstrate that the therapeutic efficacy of AURK inhibitors is highly dependent on timing relative to chemotherapy or radiotherapy (171). As a result, control of treatment timing and pharmacodynamic monitoring is required, which may be difficult to apply in routine clinical settings.

## 8 Future directions

Priority should be given to CC-specific clinical trials incorporating molecular stratification based on AURKA/AURKB dependency, mitotic instability signatures, and HPV-associated genomic features. Biomarker development is particularly critical, as AURKB expression alone may be insufficient to predict response. Promising candidates include phosphorylation status of Aurora substrates, spindle checkpoint integrity, genomic instability indices, and DNA damage response deficiencies.

Rational combination strategies represent a key opportunity to overcome resistance and enhance efficacy. Beyond traditional cytotoxic, PARP inhibitors, CHK1/ATR inhibitors, WEE1 inhibitors, and targeted agents that disrupt compensatory survival signaling could be used. Combining AURK inhibitors with DNA damage response inhibitors may potentiate mitotic arrest, especially in CC characterized by replication stress and defective DNA repair. These combinations require systematic validation in CC-specific preclinical models followed by biomarker-driven clinical trials.

Innovative drug delivery approaches may further improve the efficacy. Nanoparticle-based formulations, hydrogel systems, and tumor-targeted carriers offer the potential to improve intratumoral drug accumulation while minimizing systemic toxicity. Targeted delivery strategies guided by HPV-associated epitopes could be particularly advantageous in CC and warrant focused investigation.

The tumor microenvironment also represents an underexplored dimension. HPV-driven CC frequently exhibits immunosuppressive features, including PD-L1 expression and regulatory T-cell infiltration. While AURK inhibitors have demonstrated immunomodulatory effects in other tumor models, their interactions with immune checkpoint blockade in CC remain poorly characterized. Elucidating how AURKB/AURKA inhibition influences antigen presentation, cytokine signaling, and immune infiltration will be essential for designing rational immunotherapy combinations.

Finally, given the disproportionate burden of CC in low- and middle-income countries, future strategies must consider global accessibility, emphasizing cost-effective formulations, simplified dosing schedules, and biomarker-guided treatment algorithms to maximize clinical benefit while minimizing unnecessary toxicity.

## 9 Conclusion

Aurora kinases play a central role in the regulation of mitosis, chromosomal stability, and cell-cycle progression, and their dysregulation is a recurrent feature of CC driven largely by HPV oncogene activity. Accumulating preclinical and early clinical evidence supports the rationale for targeting AURKA and AURKB as part of combination therapeutic strategies rather than as standalone agents. AZD2811 (also known as AZD2811NP), an advanced nanoparticle formulation of barasertib (AZD1152), stands out as the most promising selective AURKB inhibitor due to its improved pharmacokinetics, favorable toxicity-efficacy profile, and ongoing clinical evaluation in solid tumors. However, the successful clinical integration of aurora kinase inhibitors in CC remains limited by several interrelated challenges, including tumor heterogeneity, variable dependency on specific aurora kinase isoforms, adaptive resistance mechanisms, dose-limiting toxicities, and complex treatment scheduling requirements. Resistance mediated through compensatory survival pathways and checkpoint adaptations further underscores the necessity of rational combination approaches guided by pathway biology. In parallel, the narrow therapeutic window of existing aurora kinase inhibitors such as Barasertib, Danusertib, Alisertib largely driven by hematologic toxicity, continues to limit dose intensity and durability of response, particularly when combined with cytotoxic chemotherapy.

Taken together, these considerations emphasize that aurora kinase inhibition in CC should be viewed not as a universal strategy, but as a context-dependent therapeutic approach requiring careful patient selection, optimized scheduling, and biologically informed combinations. Addressing these challenges through integrated mechanistic studies, biomarker-driven clinical trial design, and improved drug delivery strategies will be essential to unlock the full therapeutic potential of aurora kinase-targeted interventions. With such advances, aurora kinase inhibitors may ultimately contribute to more effective and personalized treatment paradigms for women with CC.

## Glossary

AKI: Aurora kinase inhibitors
AKT: Ak strain transforming
ATR: Ataxia Telangiectasia and Rad3-related
ATRA: All-trans Retinoic Acid
AURKA: Aurora kinase A
AURKB: Aurora kinase B
AURKC: Aurora kinase C
BAD: Bcl-2-Associated Death Promoter
BAK: Bcl-2 Homologous Antagonist Killer
BAX: BCL2 Associated X
bcl2: B-cell Lymphoma 2
Bcl-XL: B-Cell Lymphoma-Extra Large
BIM: BCL-2-Interacting Modulator of Cell Death
BIRC5: Baculoviral Inhibitor of Apoptosis Repeat-Containing 5
BUBR1: Budding Uninhibited by Benzimidazole-Related 1
CBP: CREB-binding protein
CC: Cervical cancer
CDCA8: Cell Division Cycle Associated 8
CENP-A: Centromere Protein
CHK1: Checkpoint Kinase 1
CIN: Cervical Intraepithelial Neoplasia
CPC: Chromosomal Passenger Complex
DLG1: Discs Large Homolog 1
Dsn1: Dosage Suppressor of Nnf1
E2F: E2 promoter binding factor
E6: Early Protein-6
E6AP: E6-Associated Protein
E7: Early Protein-7
Eg5: Kinesin-5
EGFR: Epidermal Growth Factor Receptor
EMT: Epithelial-Mesenchymal Transition
ERK: Extracellular Signal-Regulated Kinase
FADD: Fas-Associated Death Domain Protein
FAK: Focal Adhesion Kinase
FGFR: Fibroblast Growth Factor Receptor
GTPase: Guanosine Triphosphatase
Hec1: Highly Expressed in Cancer 1
HNCs: Head and Neck Cancers
HNSCC: Head and neck squamous cell carcinoma
HPV: Human Papillomavirus
HR-HPV: High-Risk Human Papillomavirus
HSPGs: Heparan sulfate proteoglycans
hTERT: Human Telomerase Reverse Transcriptase
IHC: Immunohistochemistry
INCENP: Inner Centromere Protein
JAK2: Janus Kinase 2
KSP: Kinesin Spindle Protein
LMICs: Low- and Middle-Income Countries
MAD2: Mitotic Arrest-Deficient 2
MAD2L1: Mitotic Arrest Deficient 2 Like 1
MAGI-1: Membrane Associated Guanylate Kinase, WW And PDZ Domain Containing 1
MAPK: Mitogen-Activated Protein Kinase
Mcl-1: Myeloid Cell Leukemia-1
MDM2: Mouse Double Minute 2
MGMT: O6-methylguanine-DNA methyltransferase
MMEJ: Microhomology-Mediated End Joining
MMPs: Matrix Metalloproteinases
MPS: Monopolar Spindle
Mps1: Monopolar Spindle 1.
mTOR: Mechanistic Target of Rapamycin,
Ndc80: Nuclear Division Cycle 80 Complex
NDEL1: Nuclear Distribution Element-1 Like-1
NF-κB: Nuclear Factor Kappa Beta
NOTCH1: Neurogenic Locus Notch Homolog Protein 1
p300: E1A-binding protein
p53: tumor protein p53
PARP: Poly(ADP-ribose) polymerase
PDK1: 3-Phosphoinositide-dependent protein kinase 1
PDZ: PSD-95, Drosophila discs large, and the adherens junction protein, ZO-1 domain
PI3K: Phosphatidylinositol 3-kinase
PLK1: Polo-Like Kinase
pRb: Retinoblastoma
RET: Rearranged during Transfection
SAC: Spindle Assembly Checkpoint
SCRIB: Scribble Planar Cell Polarity Protein
Ser: Sereine
STAT3: Signal Transducer and Activator of Transcription 3
Thr: Threonine
TNF-alpha: Tumor Necrosis Factor-alpha
TPX2: Targeting Protein for Xklp2
TRIP13: Thyroid Hormone Receptor Interacting Protein 13
TTK: Threonine Tyrosine Kinase
UBE3A: Ubiquitin Protein Ligase E3A
WEE-1: WEE1 G2 Checkpoint Kinase
Wnt: Wingless-related integration site
XRCC1: X-ray Repair Cross-Complementing Protein 1
YAP: Yes-associated protein.
